# Practical Notes on Neuralgia and Its Treatment

**Published:** 1882-08

**Authors:** 


### Practical Notes on Neuralgia and its Treatment.

There exists no better established nor important fact than that neuralgia is a disease arising when the body is in a state of general debility. This is now more generally recognized than formerly, when pain was too often regarded as the symptom of what was termed "sthenic inflammation," to be energetically treated by low diet and depleting remedies.

As this disease is frequently mistaken for rheumatism, gout, spinal irritation, etc., and *vice versa*, it may be well to name some of the leading features of a typical case of neuralgia. 1. It occurs when general debility exists, is increased by fatigue, mental or bodily, but relieved by food and sometimes by stimulants. 2. The pain, which is sudden, darting, and excruciating, exhibits remarkable intermissions, especially in the early stages of the complaint, and the constitutional disturbance is slight, (temperature, pulse, etc., frequently normal). 3. It is usually unilateral. 4. As the disease advances tender spots (points douleureux) are formed in the course of the affected nerves.

That debilty is a prime factor in neuralgia we have but to call to our remembrance cases which constantly appear. The overworked, anæmic, badly fed girl suffering from neuralgia of the fifth, the anxious struggling man in the early years of professional life or business, the married woman weakened by childbearing or over-zealous in domestic cares, and the neuralgia of declining years, degeneration having set in, nutrition being defective. In our diagnosis we are assisted by the family history of the case, whether nervous disease in any of its varied forms has existed.

The treatment should be directed in every case towards improving the general health. Nutrition must be improved by very nourishing food, well masticated, and if stimulants are prescribed they should be given with food; pure air night and day; great cleanliness, and the use of sponging with sea-salt and water. Cod-liver oil and cream are of service, given after meals. Quinine in facial neuralgias, and also chloride of ammonium; arsenic in cases of angina pectoris; iron and strychnine in anæmic states. Bromide of potassium is useful in mild cases, where the pain is not severe, but a general nervous condition exists, with restless irritability. The subcutaneous injection of morphia, beginning with one-sixth of a grain, is the most speedy and useful remedy we possess, and is a curative agent; for it checks at once pain, and thus gives us the opportunity of carrying out all those constitutional measures for improving the general health, whilst it disturbs but little appetite and digestion, and with use a toleration is established, and appetite sometimes improved; for nothing is more apt to destroy appetite than the distress of severe pain. In chronic cases of neuralgia, a blister, not necessarily carried to the point of vesication, is often of the greatest possible service, and it is a treatment peculiarly adapted to old-standing intractable cases.

Having sketched the mode of treatment it is unnecessary to give illustrations of the ordinary cases which constantly present themselves in hospital and private practive. I therefore select from my note-book one of several successful cases where neuralgia has occurred in that period of life when a cure is rarely accomplished (some authorities say *never*)—the degenerative period.

In March, 1877, I saw, in consultation with Dr. Walker, of Wakefield, a lady aged seventy-six, who in early life had suffered severely from neuralgia of the stomach, which had been much aggravated by the treatment then in vogue of insufficient nutritive food and depleting remedies. This patient was seized with violent pain, affecting the nerves of the scalp, and which became so excruciating as to deprive her of sleep for many successive nights. She became delirious in consequence, and we decided to inject one-quarter of a grain of morphia. This gave prompt relief and procured sleep. She was orderad turtle-soup, oysters, and an exceedingly nutritious dietary. She was well supplied with food at night also, which invariably relieved the pain. A mixture, containing half-drachm doses of aromatic spirit of ammonia and fifteen minims of tincture of nux vomica, seemed greatly to improve the appetite, which became prodigious and surprising. The tendency to degenerate was kept prominently in view, pure air was freely supplied in the bedroom, and every other measure taken to improve nutrition and the general health. As a local application, the chloroform liniment with tincture of opium relieved pain, and as soon as the case became chronic, the hair was cut closely and blistering fluid applied to the tender spots, which well developed in this case; multiple abscesses formed, and were frequently opened by Dr. Walker. This old lady, after an illness of three months' severe suffering, recovered perfectly, left Wakefield for Harrogate, and is now (1882) in fair health, having had no return whatever of her former complaint. Her body is feeble, but her mind extraordinarily clear and bright for a lady who has passed her eighty-first year.—*London Lancet.*

